# Case Report: Spontaneous recanalization of left anterior descending artery occlusive lesion with OCT-guided drug-coated balloon therapy

**DOI:** 10.3389/fcvm.2024.1391571

**Published:** 2024-11-20

**Authors:** Bangguo Yang, Mengqi Yeh, Jie Bai

**Affiliations:** ^1^ Department of Cardiology, Affiliated Cardiovascular Hospital of Kunming Medical University, Kunming, China; ^2^ Department of Cardiology, Fuwai Yunnan Hospital, Chinese Academy of Medical Sciences, Kunming, China

**Keywords:** acute myocardial infarction, spontaneous recanalization, occlusive lesion, optical coherence tomography (OCT), honeycomb-like structure, drug-coated balloon

## Abstract

In instances where a patient with acute myocardial infarction (AMI) did not undergo immediate reperfusion therapy during the acute phase, there was a risk of the occlusion progressing to chronic and the chances of spontaneous recanalization decreasing. This case report detailed the experience of a 37-year-old male patient who, 45 days post-AMI, still had a blocked left anterior descending (LAD) artery due to the patient's refusal for intervention. Two years later, a follow-up coronary angiography showed spontaneous recanalization of the LAD artery, with haziness in the middle segment. Optical coherence tomography (OCT) revealed a honeycomb-like structure in the mid-LAD with a minimum area of 0.55 mm^2^. The lesion was effectively treated with a drug-coated balloon, resulting in an excellent outcome.

## Introduction

Acute ST-segment elevation myocardial infarction (STEMI) is caused by plaque rupture, platelet aggregation, and activation in the coronary arteries, resulting in the sudden blockage of the blood vessels. Promptly opening the affected vessel is crucial in the treatment of this condition as it restores blood flow to the affected area and helps reduce the size of the myocardial infarction. Currently, various guidelines recommend emergency percutaneous coronary intervention (PCI) as a primary treatment option to quickly open the blocked blood vessels and restore normal blood flow, preferably achieving TIMI flow grade 3. During angiography, it has been observed that approximately 10%–30% of STEMI patients experience spontaneous recanalization (SR) of the blocked coronary arteries (1). However, without timely revascularization, the likelihood of SR decreases over time, increasing the risk of developing chronic occlusive lesions. This article presents a case study on the diagnosis and treatment of a patient who experienced SR 2 years after occlusion of the left anterior descending (LAD) artery, which was treated with a drug-coated balloon(DCB).

## Case presentation

A 37-year-old male patient with a history of smoking presented with sudden chest pain while playing basketball on January 4, 2021. The pain was located in the precordial area and accompanied by palpitations and chest tightness. There was no obvious sweating. The symptoms persisted until around 04:00 on January 5. Subsequently, the patient sought medical attention at a local hospital and underwent a comprehensive examination, which diagnosed acute anterior wall myocardial infarction (specific details unknown). Following medication treatment, the patient was transferred to a higher-level hospital for further care. At the higher-level hospital, the patient received symptomatic supportive treatment including dual antiplatelet therapy (aspirin 100 mg once daily, clopidogrel 75 mg once daily), atorvastatin, and furosemide tablets. However, the patient declined intervention treatment and was discharged with medication after showing improvement in the condition.

**Figure 1:**
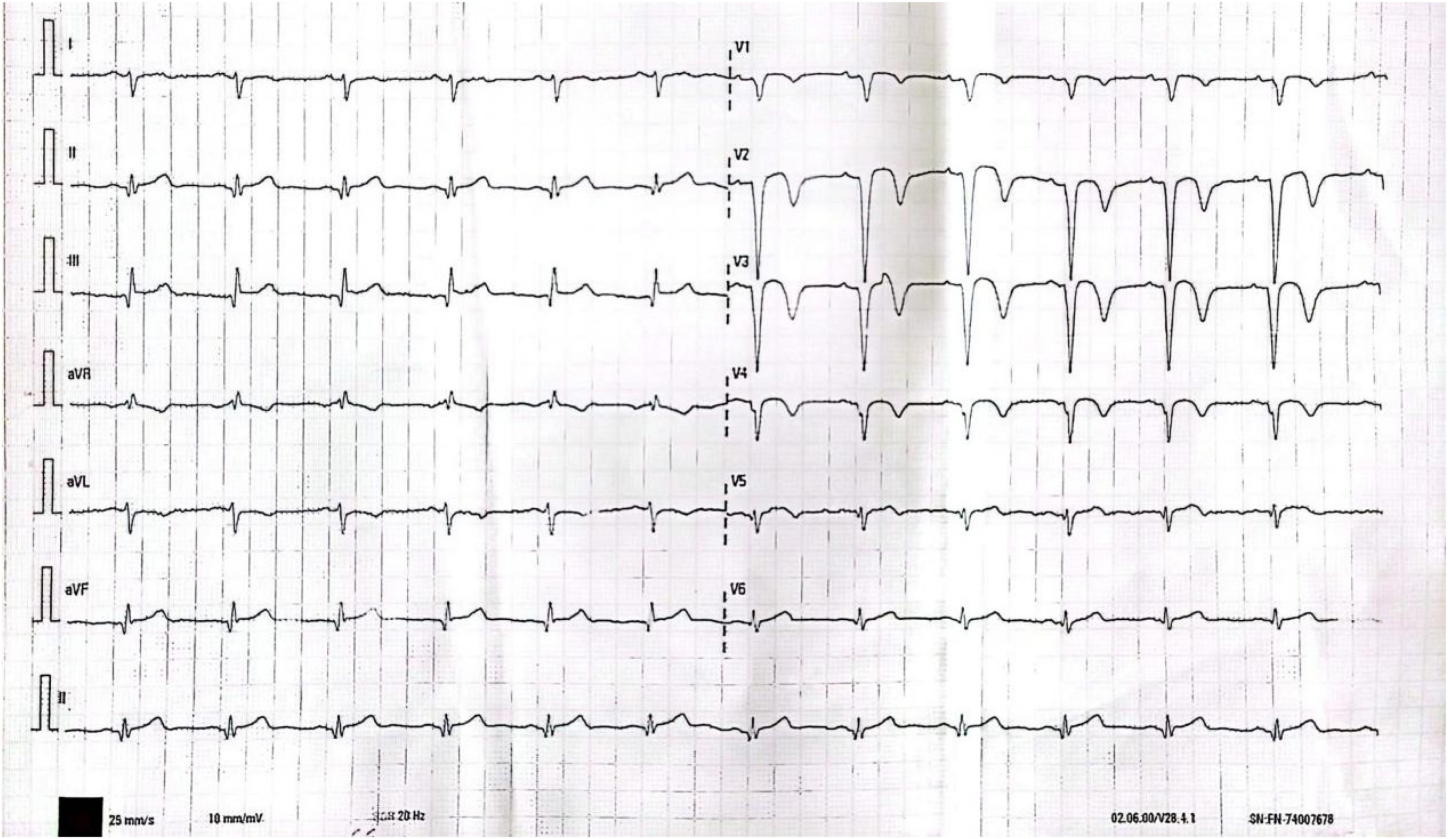
T wave inversion in leads V1-4 on 12-lead ECG.

**Figure 2:**
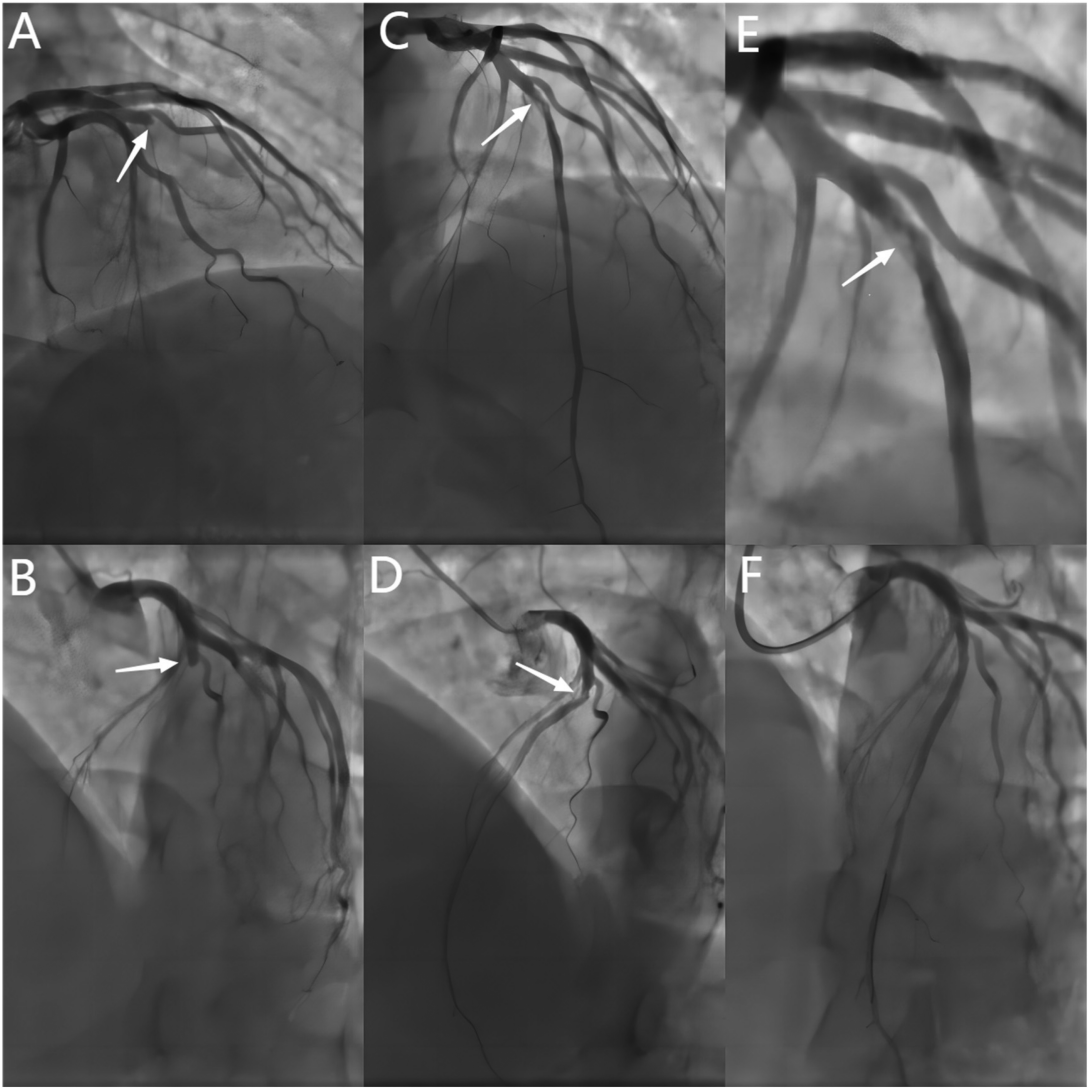
Baseline and post-procedural angiography. **(A,B)** Baseline CAG showing a 100% stenosis in the mid-LAD(white arrows). **(C–E)** Spontaneous recanalization of the LAD vessel after 2 years, with angiography showing 80% stenosis and haziness in the mid-LAD (white arrows). **(F)** Final angiographic result of DCB treatment. CAG = coronary angiography.DCB = drug-coated balloon.

After 45 days, the patient continued to experience chest tightness, chest pain, and discomfort following physical activity, prompting him to seek treatment at our hospital on February 19, 2021. Upon admission, the patient's blood pressure (BP) was measured at 96/57 mmHg, with a heart rate(HR) of 88 beats/min. No other abnormalities were detected during the physical examination. The electrocardiogram (ECG) indicated sinus rhythm, with T-wave inversion in leads V1-4 (Figure 1). Negative results were obtained from troponin and creatine kinase-myocardial band isoenzyme tests. Echocardiography revealed a left atrial anterior posterior diameter of 37 mm, a left ventricular end diastolic diameter of 46 mm, and a left ventricular ejection fraction (LVEF) of 58%. Additionally, thinning of the ventricular wall at the apex of the left ventricle and enlargement of the left atrium were observed. The following day, selective coronary angiography (CAG) revealed a 100% stenosis in the middle segment of the LAD, while no significant disease was found in the left main stem and left circumflex branch (Figures 2A,B). However, due to the patient's refusal to undergo further intervention, the coronary artery was not opened. Subsequently, the patient was discharged and prescribed dual antiplatelet medication (aspirin/clopidogrel tablets).

**Figure 3:**
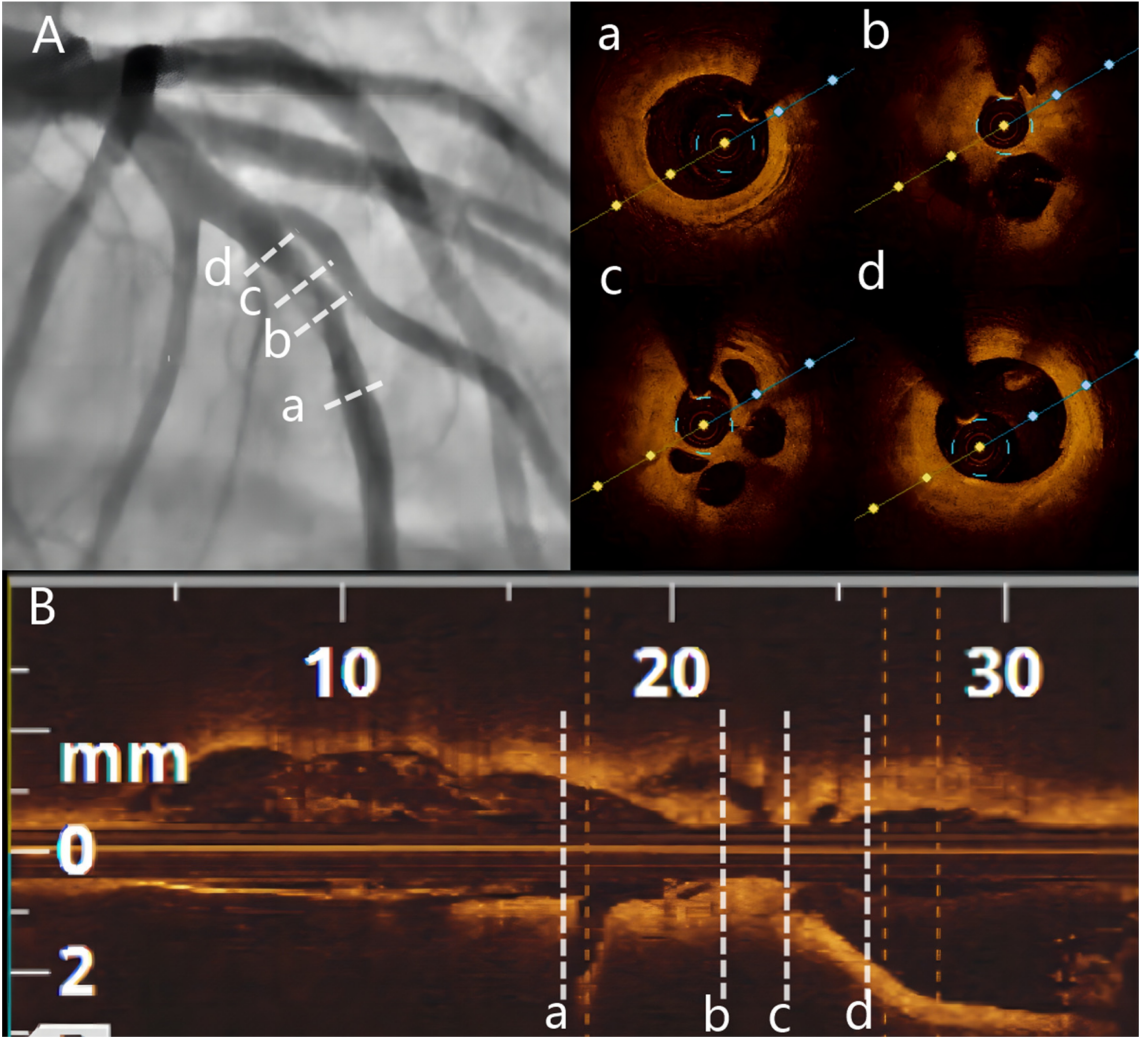
Baseline angiography and OCT. **(A)** Baseline CAG showing 80% stenosis and haziness in the mid-LAD. **(B)** Longitudinal image of OCT. (a) Distal segment of the target lesion. (b and c) OCT showing multiple honeycomb-like structures and organized thrombus shadow. (d) Proximal segment of the target lesion. OCT = optimal coherence tomography.

**Figure 4:**
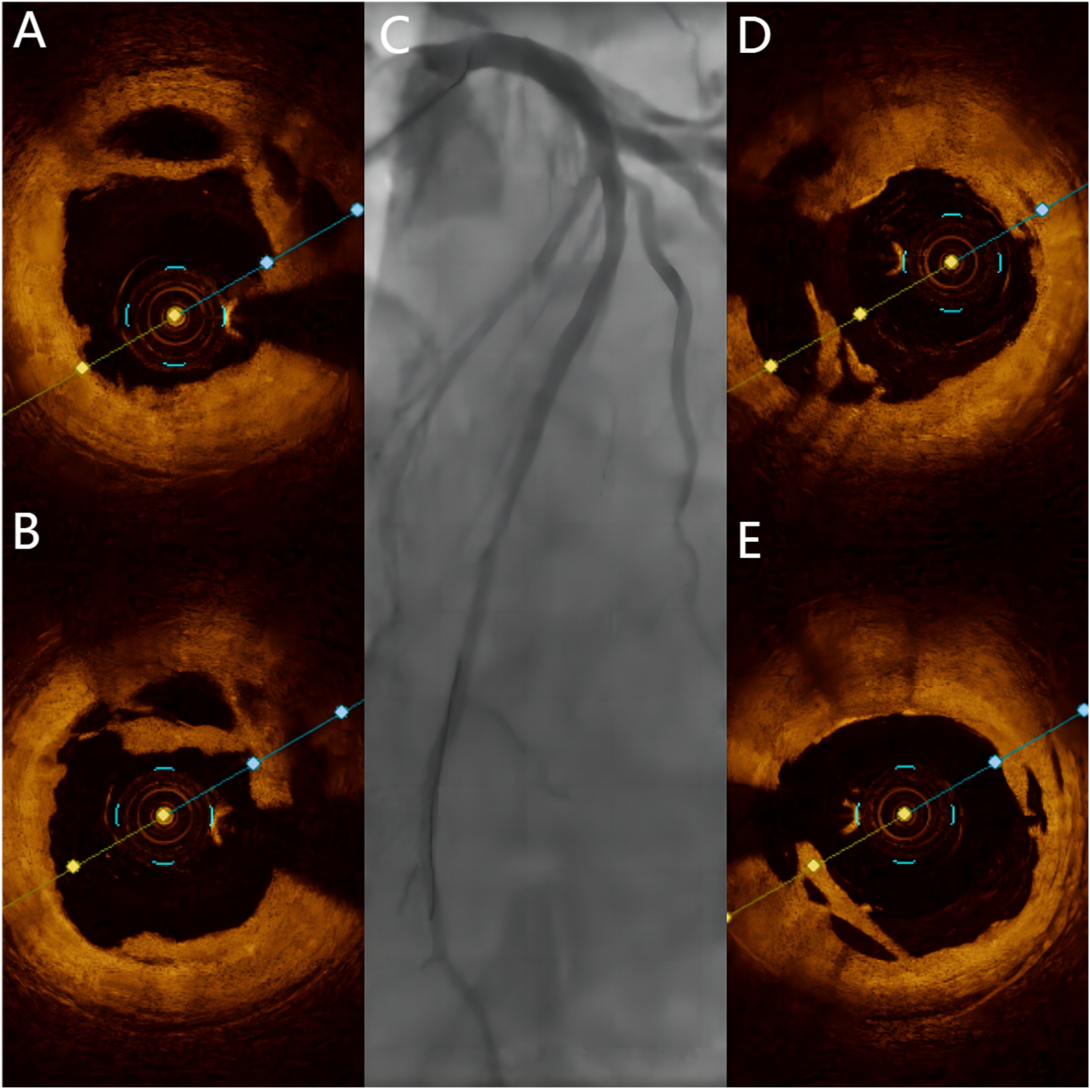
Post-Procedural angiography and OCT. **(A,B)** OCT showing honeycomb-like structures and thin fibrous septa opened by a cutting balloon. **(C)** Final angiographic result of DCB treatment. **(D,E)** Final OCT images of DCB treatment.

Two years later, the patient returned with complaints of fatigue and discomfort. Upon admission, the physical examination revealed a BP of 117/77 mmHg and a HR of 84 beats/min, with no specific symptoms. The ECG upon admission showed sinus rhythm, with a visible QS pattern in leads V1-4. The laboratory assay did not reveal any abnormal values. Echocardiography showed thinning of the ventricular wall at the apex of the left ventricle, with an estimated LVEF of 54%. On March 4, 2023, selective CAG revealed 80% stenosis and opacification in the middle segment of the LAD with TIMI flow grade 3 (Figures 2C–E).The patient underwent interventional treatment, and optical coherence tomography (OCT) examination showed a honeycomb-like structure with a minimum area of 0.55 mm^2^ in the LAD middle segment (Figures 3Ba–d). The lesion was prepared using a cutting balloon with Lepu 2.75mm × 10 mm and dilated at 8atm (1atm = 101.325 kPa). Repeat imaging revealed a residual stenosis of less than 30% (Figures 4A,B), and the lesion was further dilated with a Lepu 2.75mm × 24 mm DCB (8atm, 90s). The final angiographic result was satisfactory, demonstrating TIMI grade 3 blood flow (Figure 4C). OCT showed excellent luminal dilatation without underlying hematoma or intimal dissection, with a minimum luminal area of 4.5 mm^2^ (Figures 4D,E). The patient's condition remained stable after the procedure, and there were no further overt symptoms of angina. The patient did not experience any adverse cardiovascular events from discharge to February 2024.

## Discussions

Hazy lesions observed on CAG can be caused by fresh thrombus, calcification, spontaneous coronary dissection, ulcerated plaque, or a combination of these factors. It is important to note that coronary thrombotic tissue is increasingly recognized as a cause of these hazy lesions, as was the case in this instance. When a coronary artery becomes blocked due to a thrombus (possibly resulting from plaque rupture or spontaneous dissection), timely thrombolysis or interventional treatment is necessary to restore blood flow. Failure to do so may result in the artery remaining blocked for a long time (forming a chronic total occlusion) or undergoing partial resorption over several weeks to months, leading to the formation of a complex structure consisting of interconnected sacs and channels separated by thin fibrous septa. Autopsy studies have shown that recanalized thrombi are commonly found in patients with coronary thrombosis, with one series reporting their occurrence in up to 40% of cases (2).

This patient is the first case of SR of a coronary artery found after an interval of 2 years. The patient's angiography still showed complete vascular occlusion 45 days after the onset of the disease. No further intervention was performed due to the patient's personal reasons. But as time goes by, the thrombus is partially absorbed, forming microchannels. Under the action of antiplatelet drugs, the thrombus-rich lumen is further absorbed and dissolved, and eventually the blood vessel is gradually recanalized. The lesion appeared as a blurry image under angiography, and was eventually confirmed to be organized thrombus tissue by OCT, which appeared as honeycomb, lotus root, or Swiss-cheese-like on OCT. Whether such lesions require PCI should be determined not only by the angiographic severity of the lesion but also by the presence of symptoms and/or evidence of reversible myocardial ischemia. In the study of TianXu et al., among 16 patients with recanalization of honeycomb thrombus, 9 patients had TIMI grade 3 blood flow, but only 1 patient had normal fractional flow reserve (FFR), and the remaining patients all showed ischemia (FFR<0.8) (3). Relying solely on angiographic severity to determine the need for PCI is misleading because it correlates poorly with symptoms. Souteyrand et al. (4) found that thrombotic spontaneous recanalization lesions ranged from 11% to 100% stenosis in quantitative coronary analysis, but all patients had symptoms of angina/dyspnea and/or had reversible evidence of ischemia on functional testing. Given their highly complex morphology, one can imagine the technical difficulty of managing such lesions using guidewires, balloons, stents, or intravascular imaging devices. The study by Lee T et al. described about OCT-guided stent-less PCI with DCB for *de novo* coronary artery lesions (5). In this case, guidewire passage and subsequent advancement of the OCT catheter were easily accomplished. In view of the insufficient lumen area of this patient, combined with the characteristics of the patient's disease, a cutting balloon was used to cut the thrombus, and then a drug balloon was used for treatment. The final effect was better. Treatment of these lesions with drug-eluting stents (6), drug-eluting balloons (7, 8), and bioresorbable stents (9) has been described previously, usually with good short- and long-term outcomes. However, after stent implantation, it can damage and compress the fibrous diaphragm, potentially leading to occlusion of large branches and causing serious adverse cardiovascular events. Therefore, DCB therapy for such lesions may be an appropriate treatment option.

## Limitations

It should be noted that this study is not without limitations. (1) The patient lacked cardiac magnetic resonance data, which would have enabled the identification of the extent of scarring resulting from an acute myocardial infarction and the monitoring of disease progression. (2) Unfortunately, spontaneous recanalisation was observed only two years after LAD vessel occlusion, and the precise timing of LAD vessel recanalisation remains uncertain. A retrospective study conducted at an earlier date might have provided a definitive answer. (3) Lack of long-term coronary angiographic follow-up of the patient, although the patient had been advised to undergo coronary angiographic review approximately one year after the intervention, the patient subsequently refused further investigations due to the absence of any anginal symptoms.

## Data Availability

The raw data supporting the conclusions of this article will be made available by the authors, without undue reservation.
